# Evaluation of Teriparatide for Treatment of Osteoporosis in Four Patients with Cystic Fibrosis: A Case Series

**DOI:** 10.1155/2014/893589

**Published:** 2014-03-06

**Authors:** Oranan Siwamogsatham, Kelly Stephens, Vin Tangpricha

**Affiliations:** ^1^Department of Pediatrics, Samitivej Srinakarin Hospital, Bangkok Hospital Group, 488 Srinakarin Road, Suanluang, Bangkok 10250, Thailand; ^2^Division of Endocrinology, Metabolism and Lipids, Department of Medicine, Emory University School of Medicine, 101 Woodruff Circle NE, WMRB 1301, Atlanta, GA 30322, USA

## Abstract

*Introduction.* Bone disease is a common complication of cystic fibrosis (CF). To date, there have been no reports on the effectiveness of teriparatide, recombinant human parathyroid hormone, to treat CF-related bone disease. *Case Presentation.* We report on four patients with CF-related bone disease who were treated with teriparatide. Three patients completed two years of therapy with teriparatide, and all had significant improvements in their bone mineral density (BMD). One patient was unable to tolerate teriparatide and discontinued treatment 1 week into therapy. *Conclusion.* Teriparatide may be a potential treatment option for CF-related bone disease. This report highlights the need for further investigation into the use of teripartide in the CF population.

## 1. Introduction

Cystic Fibrosis (CF), caused by mutations in the cystic fibrosis transmembrane conductance regulator (CFTR) protein, is a common lethal genetic disease among Caucasians. As survival has improved, new complications from CF have emerged, including CF-related bone disease [1–3]. The major contributing factors to bone disease in CF include vitamins D and K malabsorption, poor nutritional status, physical inactivity, chronic inflammation, glucocorticoid therapy, delayed puberty, and hypogonadism [4–6]. These factors result in decreased bone mineral density (BMD), osteopenia, osteoporosis, fragility fractures, and kyphosis, which can cause significant morbidity and potential exclusion from lung transplantation candidacy [7]. Bisphosphonates, antiresorptive agents, have been shown to be efficacious in treating CF-related bone disease and currently are the mainstay of treatment. Studies on the use of teriparatide, recombinant human parathyroid hormone, in CF-related bone disease have not been reported to date.

## 2. Case Presentation

We report on four patients with CF-related bone disease who were treated with teriparatide 20 mcg subcutaneously once a day for a two-year period at the Emory University Cystic Fibrosis clinic. The study was approved by IRB at Emory University and all patients provided written consent for presenting their data. Demographic characteristics of our patients are displayed in Table 1.

**Case 1**

Patient 1 was a 59-year-old Caucasian female with a two-year history of CF who was referred to our clinic for evaluation of osteopenia. She was diagnosed with adult onset CF based on clinical findings after she developed a pulmonary mycobacterium avium complex (MAC) infection that was very difficult to clear. She reported being treated with prednisone two times in the past for CF exacerbations. Since menopause at 52 years of age, she had been treated with raloxifene for osteoporosis prevention. She also was on daily calcium and vitamin D supplementation with 1,200 mg and 800 international units, respectively, with a recent 25-hydroxyvitamin D (25(OH)D) level of 48 ng/mL. At the time of her referral to our clinic, her BMD revealed a T-score of −1.6 at the lumbar spine, −2.2 at the left femoral neck, and −1.6 at the total left hip. She was then started on teriparatide 20 mcg subcutaneously once a day. After 2 years of treatment, her BMD increased by 7.2% at the lumbar spine, but the total left hip was unchanged. She did not experience any falls or develop new fractures. No adverse effects from teriparatide therapy were reported.

**Case 2**

Patient 2 was a 35-year-old Caucasian female with CF (homozygous ΔF508 mutation) and CF-related diabetes who was referred to our clinic for evaluation of osteoporosis. She had been diagnosed with osteoporosis one year prior and treated with alendronate 70 mg weekly for approximately 11 months. At the time of presentation to our clinic, she had been off bisphosphonates therapy for 2 months. Her history was significant for three fragility fractures in the past five years (two rib fractures and one sacral fracture). In addition to 1,000 mg of calcium twice a day and 2,000 units of vitamin D daily, she had been on chronic steroids for the past two years for the treatment of allergic bronchopulmonary aspergillosis. She reported having normal puberty with menarche at age 12 and was on oral contraceptive pills containing estrogen. Her most recent serum 25(OH)D level was 56 ng/mL, and her BMD demonstrated a *Z*-score of −1.6 at the lumbar spine, −1.9 at the left femoral neck, and −2.5 at the left total hip. Teriparatide 20 mcg subcutaneously once a day was initiated and after completing two years of therapy, her BMD increased by 11% at the lumbar spine and by 10.2% at the left total hip. During the treatment period with teriparatide, she did report another rib fracture that occurred after excessive coughing. No adverse effects specific to teriparatide therapy were reported.

**Case 3**

Patient 3 was a 31-year-old Caucasian male with CF (2184delA/R553 mutation) referred to our clinic for evaluation of osteoporosis and vitamin D deficiency. He had been diagnosed 1 year prior with osteoporosis and was started on alendronate therapy, but he was noncompliant with treatment after one month. He was consistently taking 1,000 mg of calcium per day and 50,000 units of vitamin D twice monthly. His most recent 25(OH)D level was 33 ng/mL. He did not have a history of fractures or bone related pain; however, he had a significant decline in his hip BMD over the past 2 years. His right hip total T-score was −3.1, a 6% decrease from two years prior. He also had significant worsening of his T-score at the left hip of −2.6, representing a decrease of 5% in two years and an overall decrease of 7% from baseline. His T-score at the lumbar spine was −2.1. Given the rapid decline in his BMD, teriparatide was initiated. After 2 years of teriparatide treatment, our patient had stabilization of his hip BMD with no further significant bone loss (despite being still in the osteoporotic range). In addition, his BMD at the lumbar spine increased by 7.6%. He tolerated teriparatide therapy well with no reported adverse effects.

**Case 4**

Patient 4 was a 48-year-old Caucasian female with CF due to R117H/R560T mutation. She was referred to our clinic for evaluation of osteopenia and a decline in her hip BMD. She was a very avid runner, running approximately 4 miles a day. She did not have any history of falls, fractures, or bone pain. She was starting to have some irregular menstrual periods and hot flashes from perimenopause. She took a multivitamin on a regular basis. Her serum 25(OH)D level upon referral to our clinic was 47 ng/mL. Over the past 2 years, our patient's hip BMD had decreased approximately 4.5% with a T-score of −1.5 at the left femoral neck. Her BMD at the lumbar spine remained within the normal range with a T-score of −0.7. Given the rapid decline in her BMD in 2 years, we decided to start her on teriparatide. Soon after initiating therapy, the patient self-discontinued teriparatide due to extreme nausea. She was then started on alendronate 70 mg weekly but stopped it 1 week later due to severe myalgias. Currently she is maintained on calcium and vitamin D supplementation only.

## 3. Discussion

It is well established that bone disease is a common and major complication for individuals with CF. The prevalence of osteopenia, osteoporosis, and radiological fractures in adults with CF has been reported to be 34–38%, 13–27%, and 19–35% [8–11]. Fracture rates have been reported to be approximately twofold higher in adults with CF as compared with the general population [12]. Moreover, the prevalence of bone disease in the CF population increases with age and has been correlated with severity of pulmonary disease and nutritional status [13, 14]. As such, recommendations for screening and treatment of CF-related bone disease have been developed [15–18]. Bisphosphonates have been widely used and have been shown to be efficacious in treating bone disease in CF [19–23]. Despite the promising effects of bisphosphonates, concerns about the long-term safety and tolerability of this medication still exist [16, 24]. Potential problems with oral bisphosphonates therapy in the CF population include poor absorption leading to ineffective therapy in some patients and concerns of higher incidence of erosive pill esophagitis [16]. Furthermore, intravenous administration of bisphosphonates has been associated with severe bone pain and flu-like symptoms in CF patients, which could adversely impact the nutritional and pulmonary status of patients with CF [16]. Thus, alternative treatment strategies in this high-risk population are of great interest.

Teriparatide, a recombinant form of human parathyroid hormone, has been approved as a bone formation agent for use in osteoporotic postmenopausal women [25, 26]. This medication has been shown to increase BMD and reduce fracture rate by increasing the production of new bone via anabolic effects on osteoblasts [27]. Randomized controlled trials evaluating teriparatide and bisphosphonates therapy in postmenopausal women with osteoporosis have suggested that teriparatide is effective in increasing BMD and reducing fracture risk [28]. However, there is no data regarding the effectiveness of teriparatide in patients with CF.

In the three out of four patients who successfully completed therapy with teriparatide for CF-related bone disease, significant improvements in BMD were seen after 2 years of therapy. These findings suggest that anabolic therapy for bone disease in the CF population may be an effective and safe treatment modality, particularly in those who are intolerant of bisphosphonates. One patient was intolerant of the teriparatide but she was also intolerant of bisphosphonates. Given these promising results in these 3 patients, further studies are warranted to evaluate the efficacy and safety profile of recombinant human parathyroid hormone analogue therapy in the CF population.

## 4. Conclusion

In patients with CF-related bone disease, teriparatide may be a potential treatment option. This report highlights the need for further investigation into the use of teriparatide in the CF population.

## Figures and Tables

**Table 1:** Demographic data.

	Patient 1	Patient 2	Patient 3	Patient 4
Age (year)	59	35	31	48
BMI (kg/m^2^)	19.6	21.8	20	18.5
Ethnicity	Caucasian	Caucasian	Caucasian	Caucasian
Gender	Female	Female	Male	Female
Gene mutation	—	ΔF508 Homozygous	2184delA/R553	R117H/R560T
Baseline 25(OH)D (ng/mL)	48	56	33	47
BMD (T-score)				
L1–L4	−1.6	−1.6*	−2.1	−0.7
Left femoral neck	−2.2	−1.9*	−1.9	−1.5
Left total hip	−1.6	−2.5*	−2.6	—

*indicates *Z*-score.

## Abbreviations

CF: Cystic fibrosis
BMD: Bone mineral density
CFTR: Cystic fibrosis transmembrane conductance regulator
MAC: Mycobacterium avium complex
25(OH)D: 25-hydroxyvitamin D.
